# Health Tracts

**Published:** 1867-04

**Authors:** 


### HEALTH TRACTS.

We have written a volume of Health Tracts, some three hundred in number on a great variety of subjects. Sent post-paid for $2.50, by addressing "The publisher of Hall's Journal of Health, No. 2 West 43d Street, New York;" it is full of such instructions as all households need and no young person can fail to obtain from it most valuable information as to the preservation of health and the prevention of disease, but there are many who do not feel able to purchase the entire volume, but yet would like to possess some special tract, for the convenience of such the following contents of each month are appended; any number is sent post-paid, for fifteen cents, by addressing as above; any two numbers sent for twenty-five cents.

JANUARY.—Inconsiderations; Summer Fruits; How to Cure a Cold; Burying Alive; Care of Eyes; Traveling Hints; Young Old People; Drunkenness and Dyspeptia; Uses of Ice; Winter Rules; Walk Erect; How to Wash; How to sit; Winter Shoes; Growing Beautiful; Measles.

FEBRUARY.—Sabbath Physiology; Hair Treatment; Wearing Flannel; Health without Medicine; Cold Feet; Costiveness; Bites and Burns; Sour Stomach; Sleeping; Eating, Bites, Corns and Odors. Advertising Disease; Neglected Colds; Bathing; Colds Avoided; Eating Wisely; Dyspeptia; How to Eat; Drinking; "Nothing but a Cold;" Precautions; Healthful Observances.

MARCH.—Presence of Mind; Sick Head Ache; Health's Three Essentials; Premonitions; Neuralgia; Private Things; Coffee Drinking; Success in Life; A Warning to Youth; Hydrophobia; Rheumatism; Sunshine; Catarrh; 15 Follies; Diet for Invalids; Poisons and Antidotes; Erysipelas; Nursing Hints; Apples Healthful.

APRIL.—CHECKING PERSPIRATION (the most important tract of the 300;) Taking Medicine; Failing Eyesight; Head Ache; Skating; Inverted Toe-Nail. Physiological; Urination; Pain; The Teeth; Read and Heed; Deafness; Nervous Sufferers; Coffee Substitutes; Best Supper; Beards; Housekeeping; How to Live Long; Health Theories; Vaccination.

### SUMMERINGS.

MAY.—Scalds and Burns; Music Healthful; Milk; Getting a Living; Corn Bread; Sabbath Rest; Dieting; Woman's True Beauty; Debt a Death; Law of Love; Soldiers Remembered; Spinal Disease; Rearing Children; Child Bearing; Housekeeping Hints; Duration of Life; Serenity; Diarrhoea; Miasm and Fever and Ague.

JUNE.—Habit; Soldiers Cared For; Sores; Greed of Gold;

Preserving Fruits; Sunday Dinners; Marriage; Beauty a Medicine; Small Pox; Parental Training; Whitlow or Felon; Sleep and Death; Soldier Health; Soldier Items; Morning Prayer; Fire-places; Apples Kept; Consumption; Soldiers All; Children's Eating.

JULY.—THAT BEST DAY; Coal Fires; Deafness; Housewifery Potatoes; Badness; Ventilating Theories; Cute Things; The One Spot; Coffee Poisons; Burning to Death; Woolen Clothing; Whitewashes; School Children.

AUGUST.—Life Wasted; Poisons and Antidotes; Cures; Household Vermin; Philosophy; Specifics; One Acre; Spring Time; Changing Clothing; Eating Habits; Dying Easily; Catching Cold; Resignation; Growing; Dieting not Starvation; Summer Drinks; Diptheria; Diarrhoea; Cholera; Dysentery.

SEPTEMBER.—Bilious Diarrhoea is Curative; Disinfectants; Physiological Items; Sabbath Observance Healthful; Five Escapes; Saving Ministers; Death Rate; One by one, our friends pass away; Vices of Genius; Leaving Home; Fifth Avenue Sights; Sickness not Causeless; Obscure Diseases; The Benevolent Banker; Worth Remembering; Physiology of Public Worship; Medical Items; The Month Malign; Great Eaters; Logic Run Mad; Insanity; Philosophy of Exercise; Summer Mortality; Posture in Worship.

OCTOBER.—Cancer; Weather Signs; Stammering; Children's Feet; Gruels and Soups, how made; Medical Melange; Sick School Girl; Kindness Rewarded; Mush and Milk; Deranged correcting Children; Convenient Knowledge in the Mind; Charms; Curiosities of Eating; Mind and Body, their mutual influences; Bread; Memories; Emenations; Eating Economically; Effects of Liquor; Balm of Gilead.

NOVEMBER.—Where to Study; Weather and Wealth; Apoplexy; Stomach's Appeal; Household Knowledge; Who are Happiest; Cooking Meats; Food and Health; Inheritances; Restless Nights; Summer Recreations; Cheap Bread; Nutriment in Food; Facts about Eating; Object of Eating; Growing Potatoes; Value of different kinds of Food; Horse Rations; Hunger; Digestibility of various kinds of Food; Warmth and Strength, where desired.

DECEMBER.—Ill-Smelling Feet; Mind Lost; Elements of various kinds of Food; Worth Knowing; "Dirty" Children don't live Long; Salt Rheum; Medical Science; Popular Fallacies; Church Ventilation; Cough; Druggery; Sordid; Restlessness; Punctuality; Providence; Ever Thankful.

One number, sent free for 15c., any two for 25c., by addressing "The Publisher of Hall's Journal of Health," No. 2 West 43d street, New York, or the whole volume, containing 285 tracts, and a steel engraving, is sent, post-paid, for $2.50.

